# Digital Pathology‐Based Comparison of PyRadiomics and HistomicsTK for Nuclei Classification in Melanoma Whole Slide Images

**DOI:** 10.1155/ijbi/7419529

**Published:** 2026-04-01

**Authors:** Alessia Finti, Franco Marinozzi, Giovanni Pasini, Giorgio Russo, Alessandro Stefano, Fabiano Bini

**Affiliations:** ^1^ Department of Mechanical and Aerospace Engineering, Sapienza University of Rome, Rome, Italy, uniroma1.it; ^2^ Institute of Bioimaging and Complex Biological Systems, National Research Council (IBSBC-CNR), Cefalù, Italy; ^3^ National Laboratory of South, National Institute for Nuclear Physics (LNS-INFN), Catania, Italy

**Keywords:** artificial intelligence, digital pathology, melanoma, radiomics, whole slide images

## Abstract

**Background:**

The analysis of histopathological characteristics from biopsy whole slide images (WSI) is a standard procedure in current diagnostic workflows. For instance, malignancies such as melanoma often require the execution of biopsy to be accurately identified. However, diagnosis can be difficult because of variability in clinical scenarios and in microscopic pictures, as well as the lack of biomarkers availability. In this context, the extraction of shape, texture, and intensity‐based features from medical images has proven to be a very promising strategy to uncover latent patterns that may be helpful for diagnosis and prediction of several pathologies.

**Methods:**

This study proposes radiomics as a powerful tool for extracting nuclei features and enabling nuclei classification of PUMa dataset melanoma WSIs. More specifically, it evaluates the extraction of radiomics features through PyRadiomics, in comparison with the pathomics tool, namely HistomicsTK, in terms of classification performance. To systematically compare these approaches, three supervised classifiers were trained and tested using the same training/testing splits and usual classification metrics: one on radiomics features, one on histomic features, and one on the merged feature set.

**Results:**

The results illustrate an improved performance of the radiomics model compared with both the histomic model and the hybrid radiomics and histomics model, suggesting that radiomics can extract valuable phenotypic information from histological images.

**Conclusions:**

Radiomics‐based feature extraction, as implemented in PyRadiomics, may be a valid and robust alternative to histomics/pathomics descriptors implemented in HistomicsTK in computational pathology pipelines for melanoma analysis.

## 1. Introduction

According to the International Agency for Research on Cancer (IARC) of World Health Organization (WHO), skin cancers are the most common group of malignancies diagnosed worldwide. More specifically, in 2022 around 330,000 people were diagnosed with melanoma and almost 60,000 people died because of this pathology [1]. Although the incidence of cutaneous malignant melanoma (CMM) is rapidly increasing worldwide, CMM mortality in young adults has recently been decreasing thanks to early CMM diagnosis and prevention practices [2]. According to Boutrous [3], the treatment of advanced melanoma has recently been improved by immune‐checkpoint inhibitors and targeted therapies. On the other hand, metastatic melanoma is usually associated with poor prognosis, and survival is based on the degree and site of metastasis, that is, skin, lungs, liver, and central nervous system [4]. Metastatic melanoma is affected by a low 5‐year survival rate and, even if some improvements were recorded in the 3‐year survival rate, the persistent incidence of melanoma arises the need for more effective therapeutic approaches [5]. According to the Brazilian Society of Pathology, melanoma diagnosis can be difficult because of diversity of clinical scenarios, microscopic pictures, and lack of biomarkers availability [6]. In the diagnosis and pathology guidelines [6], the authors outline that melanoma evaluation is first carried out through noninvasive dermoscopy. If a suspicious lesion is identified, it is sampled and analyzed through excisional or incisional biopsies. Together with the examination of patient′s clinical history and conditions, microscopic examination of melanoma diagnostic features and histopathological characteristics is executed through biopsy whole slide images (WSI) analysis [6, 7]. With the heterogeneity of melanoma, there is a growing need for robust analytical tools that can assist with diagnosis, prognosis, and prediction of response to treatment.

Radiomics (RAD) is a quantitative imaging technique that seeks to provide additional information to the clinician. By automatically extracting quantitative patterns from images, RAD quantifies textural features using analytical artificial intelligence (AI) methods [8]. RAD has already proven to hold promise for the assessment of melanoma, for the prediction of the response to tyrosine kinase inhibitors (TKIs) and immune checkpoint inhibitors (ICI) immunotherapy and for predicting mutational status [9]. Furthermore, RAD applied to computerized tomography (CT) together with clinical parameters analysis has been examined to predict response and survival in patients affected by melanoma and undergoing immunotherapy [10]. In wider terms, the combination of AI and RAD applied to positron emission tomography (PET), CT or magnetic resonance imaging (MRI) holds the potential for the identification of better diagnostic and prognostic biomarkers [11]. Moreover, PET and PET/CT RAD offer unique insights into tumor biology and treatment response [12, 13].

In this context, this study presents a controlled benchmark for the use of RAD‐based feature extraction to melanoma nuclei WSIs. A comparison is also performed with a nuclei‐oriented feature extraction library under default configurations, in terms of classification performance of trained classifiers. The classification consists in discriminating between tumor nuclei and other types of nuclei, in both primary and metastatic slides. By comparing performance across different feature sets, the study is aimed at empirically identifying whether RAD‐based descriptors can provide complementary or more informative contributions for this specific WSIs melanoma nuclei analysis.

## 2. Related Work

### 2.1. Nuclei‐Level Pathomics and Morphological Descriptors

As stated in [14], since WSIs typically contain many objects in a heterogeneous histologic landscape, pathomics can be a powerful tool to classify cellular interactions through the identification of relevant spatial relationships, important for clinical outcomes and treatment response. In this context, pathomics workflows are commonly based on the definition of regions of interest (ROIs), followed by the extraction of handcrafted nuclei‐level features describing morphology, intensity, texture, and spatial organization [15]. ROI definition, which can be performed manually or automatically, ensures that extracted descriptors are representative of the underlying tissue or cellular structures and suitable for downstream classification or prognostic tasks [15].

Recent advances have emphasized the importance of accurate nuclear segmentation and classification as a prerequisite for reliable pathomics analysis. For instance, Zhang et al. [16] proposed an enhanced nuclei analysis framework that jointly segments and classifies nuclei, improving robustness to heterogeneous staining and tissue variability. Moreover, toolkits like HistomicsML2 [17] and HistomicsTK [18] have been proposed for specialized pipelines of classification of WSIs and for nuclear segmentation and shape‐based feature extraction.

### 2.2. RAD in Histopathology

Although RAD typically captures macroscopic tissue heterogeneity in radiology, its application to WSIs has recently emerged as a complementary approach. More specifically, texture RAD has been described as a powerful strategy for the extraction of quantitative features from histology images, since it captures subtle changes in tissue architecture that may not be apparent through conventional histopathological assessment [19].

Pathomics provides microscale, cell‐level information extracted from histopathology of digitalized tissue slides, complementing RAD features that typically capture macroscopic tissue heterogeneity [20]. Thus, together with pathomics, the application of RAD to WSIs appears to be an emerging tool: for example, RAD has been employed to improve nuclei segmentation from different tissues [21] and to run deep textural analysis of early breast cancer histopathological slides using an unselective RAD analysis methodology [22]. Other frameworks, such as “RadPleura” framework [23], have explored RAD for the classification of tissue regions in pleura histopathological images.

Recent studies have also highlighted the cross‐scale integration of pathomics and RAD features. Dia et al. [24] investigated the association between pathomics and RAD features in immunotherapy‐treated NSCLC patients, showing that combining microscale nuclear descriptors with macroscale tissue RAD can provide complementary information relevant for patient stratification. Similarly, in [25] it was performed an exploratory analysis in uterine corpus endometrial carcinoma, demonstrating that integrated RAD ‐pathomics analyses can enhance the identification of tumor subtypes and potentially improve prognostic modeling.

### 2.3. Deep Learning (DL) in WSIs Nuclei Analysis

Although the present study does not employ DL, several DL‐based approaches have been proposed for nuclei and WSI analysis in melanoma and other cancers. The combination of DL techniques and pathomics analysis based on cell nuclei features from WSIs has emerged as a promising approach for tissue characterization and tumor classification [26]. Beyond radiological imaging, DL algorithms have proven capable of enhancing histopathology analysis by automatically extracting discriminative features that capture complex nuclear morphology and tissue architecture. For instance, convolutional neural networks (CNNs) have been used to organize WSI images from different tissues into regional partitions that show relative cytoarchitectural uniformity [27]. Supervised learning models have also been used to classify melanomas and nonmelanomas WSIs [28].

Recent studies have explored hybrid strategies that integrate handcrafted pathomics features with DL representations, leveraging the complementary strengths of both approaches. For instance, in [29] it was demonstrated that combining handcrafted nuclear descriptors with deep features in ensemble models improves classification performance in colon cancer histopathological images. Similarly, Huang et al. [30] proposed a fusion of handcrafted and deep features in a CNN framework for prostate cancer detection, showing enhanced predictive accuracy compared with feature type alone. In melanoma histopathology [31], a DL‐based nuclei segmentation pipeline with test image augmentation and ensemble modeling was employed to improve melanoma detection from skin WSIs.

## 3. Materials and Methods

**Figure 1:**
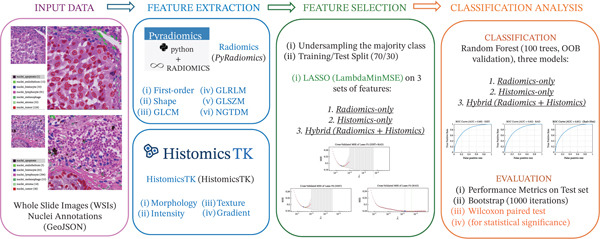
Pipeline for validating the proposal of radiomics as an alternative tool for nuclear feature extraction and tumor nuclei classification. Starting from melanoma primary and metastatic nuclei‐annotated WSIs, feature extraction is performed through pathomics and radiomics tools. After feature selection, classification is performed on three sets of features and performance is statistically compared.

To evaluate the potential of RAD as a powerful tool for nuclear feature extraction and classification, in comparison with traditional pathomics approaches, the pipeline represented in Figure 1 was developed. More specifically, starting from nuclei‐annotated WSIs of primary and metastatic melanoma, feature extraction was performed both with *PyRadiomics* [32] and Pathomics toolkit *HistomicsTK* [33] (hereafter referred to as *Histomic* [HIST] features). The extracted features were evaluated separately and together as a hybrid feature set containing both sets of features. Therefore, after dividing nuclei into tumor and nontumor classes and after verifying class balance, feature selection was conducted independently for each model: RAD, HIST, and hybrid model (RAD + HIST). Three random forest classifiers were trained on the respective sets of selected features, and their performances were evaluated and statistically compared.

### 3.1. Panoptic Segmentation of Nuclei and Tissue in Advanced Melanoma (PUMa) Dataset

This study relies on the publicly available portion of the PUMa database [34, 35]. The database is made up of hematoxylin and eosin (H&E)‐stained ROIs in *tif* format of primary and metastatic melanoma samples, each with their corresponding nuclei and tissue annotations in *GeoJSON* format. In total, the whole dataset contains 155 primary tumors ROIs and 155 metastatic tumors ROIs, all being acquired from one single specialized melanoma treatment center at 40× magnification (0.22 *μ*m/px) and 1024 × 1024 pixels resolution. As explained by Schuveling et al. [35], PUMa is the first dataset fully focused on melanoma and gives the foundation for the creation of segmentation algorithms tailored to this pathology with relevance to diagnostic and prognostic biomarker discovery. For this study, the public dataset subset of 103 primary and 103 metastatic ROIs was used, containing a total amount of 97,429 annotated and investigated nuclei. Figure 2 presents representative images of the ROIs we are investigating for both melanoma types, with annotation overlays depicted using the QuPath open‐source digital pathology software [35]. Figure 2a represents primary melanoma ROIs, respectively, annotated in QuPath Figure 2b. Metastatic melanoma ROIs are represented in Figure 2c, respectively, annotated in QuPath Figure 2d. Annotated nuclei were classified into 10 categories: *“nuclei_tumor,” “nuclei_lymphocyte,” “nuclei_plasma_cell,” “nuclei_hystiocyte,” “nuclei_melanophage,”* “*nuclei_neutrophil,” “nuclei_stroma,”* “*nuclei_endothelium,” “nuclei_epithelium,”* and *“nuclei_apoptosis”.* To enable the development of a binary classifier focused on tumor detection, all nontumor categories were grouped together into a single “non‐tumor” class.

Figure 2Example of primary (a) and metastatic (c) ROIs from PUMA dataset explored in QuPath open‐source digital pathology software. Panels (b) and (d) show annotation overlays depicted in QuPath.

**Figure:**
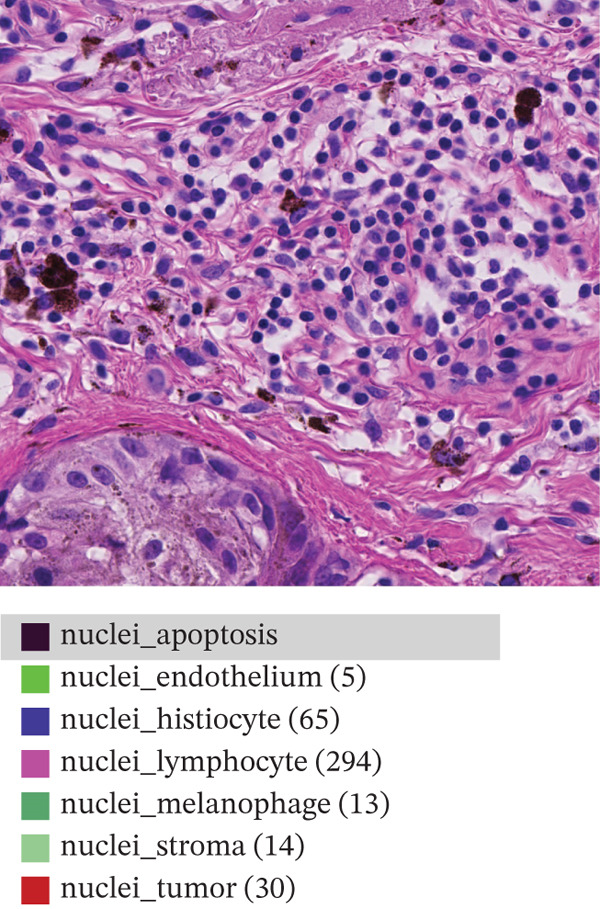
(a)

**Figure:**
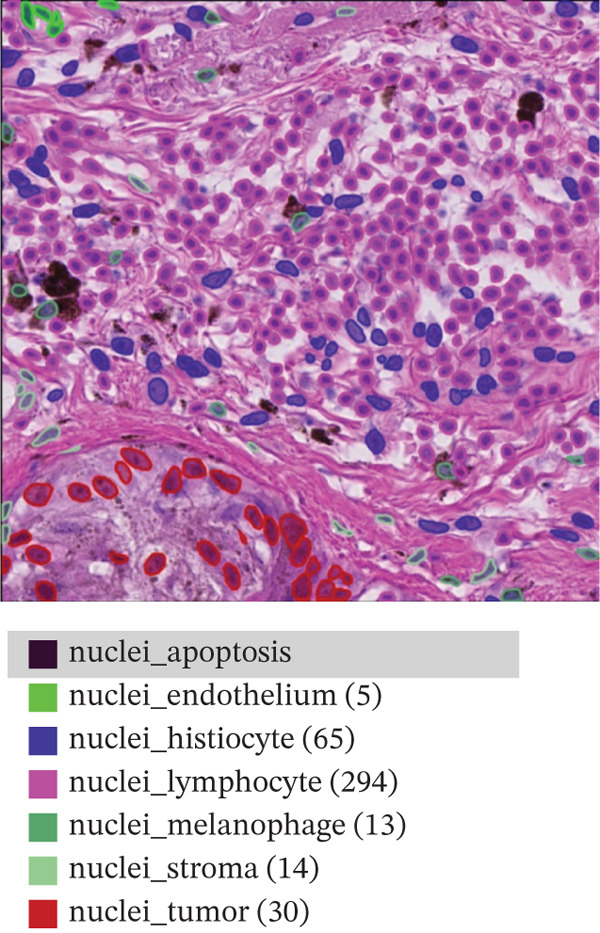
(b)

**Figure:**
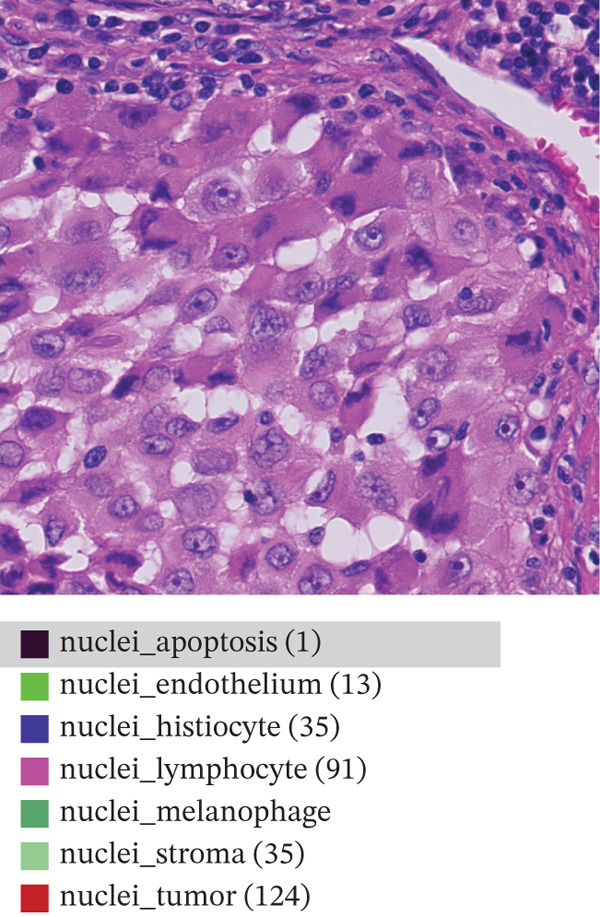
(c)

**Figure:**
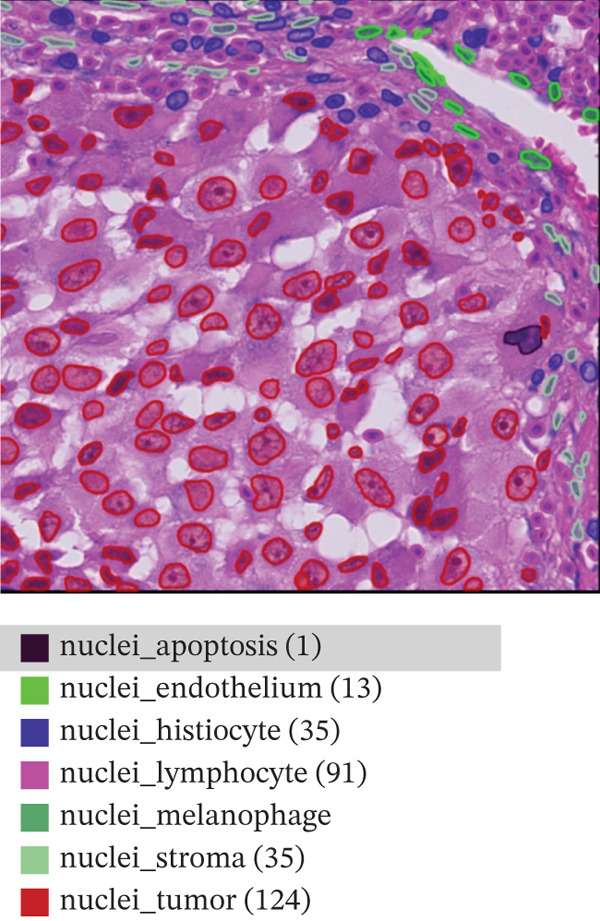
(d)

### 3.2. Extraction of RAD and HIST features

To evaluate the behavior of RAD‐based and HIST‐based feature extraction for nuclei analysis, both RAD and HIST descriptors were computed for every annotated nucleus. RAD features were extracted through PyRadiomics, an open‐source python package for the extraction of RAD features from medical imaging [32]. PyRadiomics provides five classes of features consisting of: first‐order statistics, shape descriptors, texture classes, gray level cooccurrence matrix, gray level run length matrix, and grey level size zone matrix. PyRadiomics also aligns with the Image Biomarker Standardization Initiative (IBSI) guidelines [36] and RAD features were extracted with default parameters. To reflect the default settings of the library, no additional feature families or customized parameters were introduced. HIST features were computed through HistomicsTK [33]. HistomicsTK is a Python package for the analysis of digital pathology images that can be used as a stand‐alone library or as a Digital Slide Archive plugin that enables performing image analysis through a user interface, namely HistomicsUI. More specifically, “*histomicstk.features.compute_nuclei_features*” function was chosen for the current analysis, being tailored to compute features for nuclei classification. This function was applied without modification, using its default feature set. Computed HIST features include spatial, morphometric, intensity, gradient, and textural descriptors of each nucleus [37].

### 3.3. Feature Selection and Fusion Strategy

Before the execution of dimensionality reduction, under‐sampling of the majority class (tumor nuclei) was performed in MATLAB (v. 2024(b)) [38] to avoid class imbalance. Furthermore, the dataset was split into 70% training and 30% test. The subsequent dimensionality reduction was performed only on the training set to avoid using information from the test set during feature selection. For each experiment to be consistent, a shared MATLAB *cvpartition* object was reused in all RAD, HIST, and fused feature analyses. This guaranteed that the same samples were assigned for training and testing subsets throughout, enabling the comparison among models. To reduce dimensionality and enhance the generalizability of the classification models, a multistep feature selection strategy based on LASSO (Least Absolute Shrinkage and Selection Operator) regression was applied through MATLAB′s *lassoglm* function. Feature selection was performed to simplify the subsequent training of classifiers and to avoid the use of noninformative predictors. In all analysis, LASSO regression feature identification was performed through the LambdaMinMSE criterion, the value of lambda minimizing the cross‐validated mean squared error (MSE). Three separate LASSO regressions were performed: one for RAD features, one for the HIST features, and the last for a fused feature set obtained by concatenating both RAD and HIST features. In this way, three distinct sets of selected features were obtained. Each of these sets was then used to train a corresponding random forest classifier, enabling a direct comparison in terms of performance among the three classifiers as explained in the next section. To avoid information leakage, all feature selection steps were carried out exclusively on the training data, whereas the test set was set aside and used only for the final evaluation of model performance.

### 3.4. Classification Framework and Comparative Evaluation of Feature Sets

For each of the three final feature sets, a random forest classifier was trained. Random forest classifier was selected after comparing its performance with alternative classifiers (i.e., AdaBoost and Gradient Boosting) which resulted in lower values of performance metrics. Feature values were normalized with *z*‐score normalization prior to training, with mean and standard deviation computed only from the training set to ensure consistent scaling for both training and test sets. The classifiers were trained in MATLAB through the *TreeBagger* function, instructed to generate 100 decision trees and perform out‐of‐bag estimation for internal validation. Performance of the classification models was assessed on the independent test set using a range of metrics derived from the confusion matrix, including accuracy, precision, recall (sensitivity), specificity, F1‐score, and area under the ROC curve (AUC). ROC curves were also drawn in addition to visually assessing the discriminative ability of the classifiers. To further assess the potential contribution of RAD features to the classification of the tumor nucleus, a comparative examination across RAD features, HIST features, and RAD + HIST features was performed. To introduce robustness and to account for variability due to data partitioning, the performance of the models was evaluated using bootstrap resampling. One thousand bootstrap samples were generated, and for each sample performance were derived. To assess whether the performance differences among the resulting distributions of these metrics across the three models were statistically significant, the models were compared statistically using the paired Wilcoxon signed‐rank test.

## 4. Results

### 4.1. Feature Selection and Fusion Strategy

Figures 3a, 3b, and 3c show the cross‐validation curves of LASSO fit for the RAD, HIST, and hybrid models. Each plot shows the MSE as a function of the regularization parameter Lambda. In all cases, the vertical lines (and/or the colored green and blue dot points) represent the Lambda associated with the one‐standard‐error rule (Lambda1SE) and the Lambda value that corresponds to the minimum MSE (LambdaMinMSE) which was used as the selection criteria for determining the final set of selected features. For the RAD model, 102 features were reduced to 69. For the HIST model, the original amount of 76 features was reduced to 8. For the HIST + RAD model containing 178 combined features, LASSO selected only 11 features, respectively, 6 RAD features *(“original_firstorder_10Percentile,” “original_firstorder_Minimum”original_gldm_DependenceVariance,” “original_gldm_GrayLevelNonUniformity,” “original_glrlm_RunLengthNonUniformityNormalized,” “original_glrlm_ShortRunEmphasis”*) and 5 HIST features (*“ShapeFSD4,” “ShapeFSD6,” “NucleusGradientMagStd,” “NucleusHaralickSumAverageRange”).*

Figure 3Cross‐validation outcomes of the LASSO regression for the RAD (a), HIST (b), and HIST + RAD models (c), respectively. In each plot, the mean squared error (MSE) is shown as a function of the regularization parameter Lambda. The dashed green line indicates the value of Lambda that achieves the minimum MSE (LambdaMinMSE), whereas the dashed blue line corresponds to the one‐standard‐error criterion (Lambda1SE), a more conservative threshold that promotes a more compact and potentially more stable set of selected features.

**Figure:**
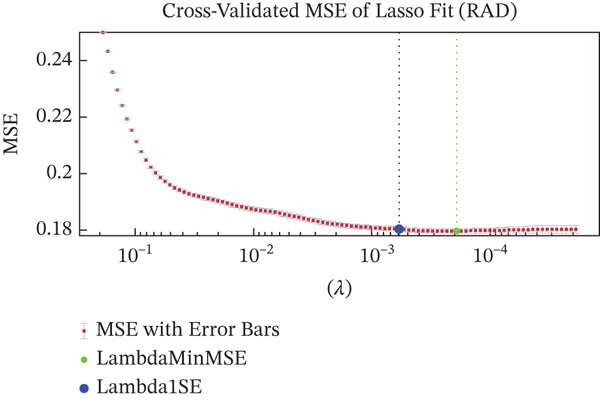
(a)

**Figure:**
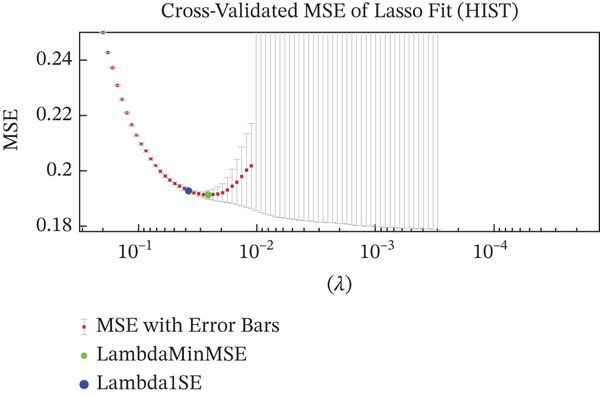
(b)

**Figure:**
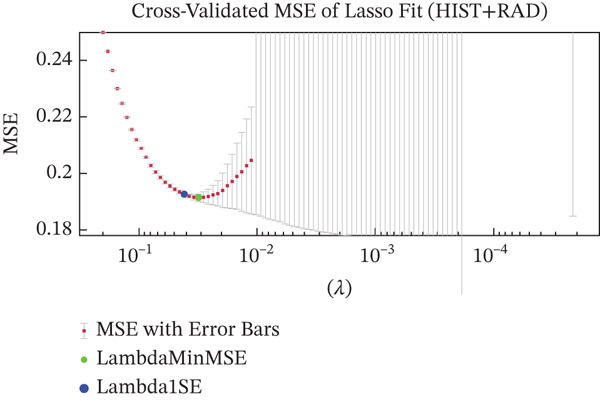
(c)

### 4.2. Classification Analysis

The performance of each classifier, trained, respectively, on RAD, HIST, and RAD + HIST feature sets, was assessed using standard classification metrics computed on the independent test set. Figure 4 and Table 1 describe the classification performances of the three models.

Figure 4Confusion matrices of the random forest classifiers trained on different feature sets. (a) Radiomics model (RAD); (b) histomic model (HIST); (c) Hybrid model combining radiomic and histomic features (HIST + RAD). The *x*‐axis indicates the predicted class (0 = nontumor, 1 = tumor), and the *y*‐axis indicates the true class.

**Figure:**
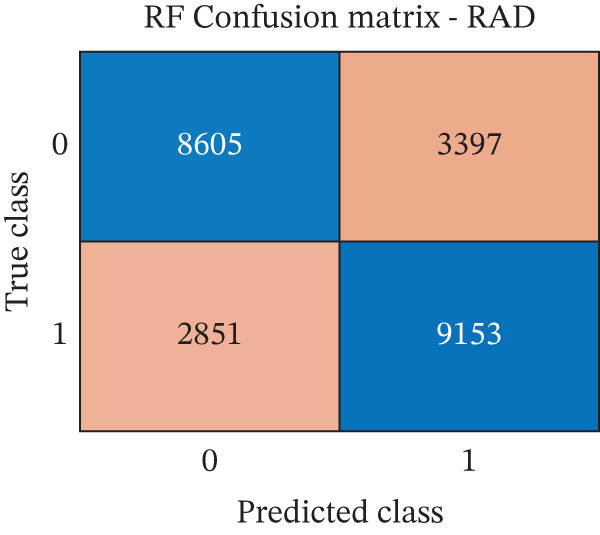
(a)

**Figure:**
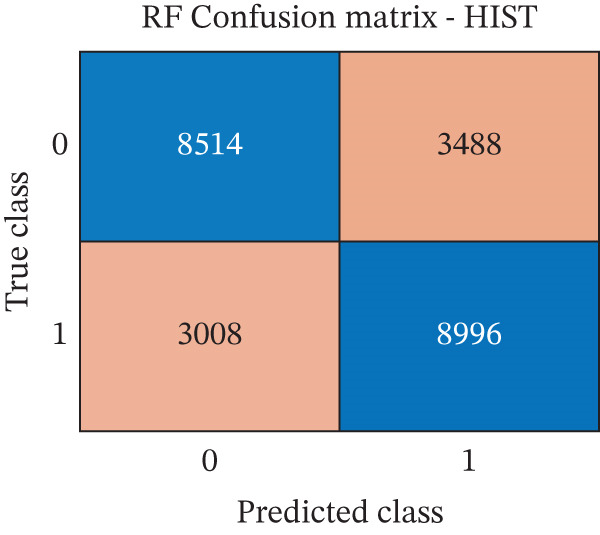
(b)

**Figure:**
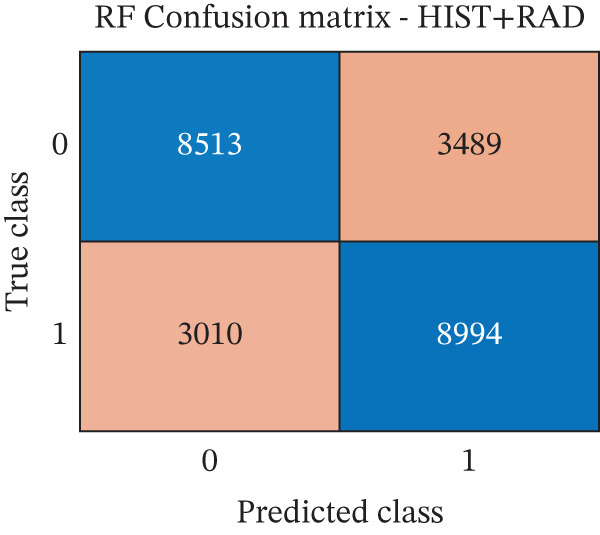
(c)

**Table 1:** Classification metrics for the three models: RAD, HIST, and HIST + RAD. Best values per metric are highlighted in bold.

Metric	Radiomic Model	Histomic model	Hybrid model
Accuracy	73.97%	72.94%	72.93%
Precision	73.0%	72%	72%
Recall	76.0%	75%	75%
Specificity	72.0%	71%	71%
F1‐score	75.0%	73%	73%
AUC	0.82	0.80	0.81

As shown in Table 1, the RAD model revealed better performances than both the HIST and HIST + RAD models across all evaluation metrics: It achieved the highest accuracy (73.97%), precision (73.0%), recall (76.0%), specificity (72.0%), F1‐score (75.0%), and AUC (0.82). The HIST + RAD model showed no significant improvement over the HIST model, indicating that the addition of RAD features did not improve performance metrics if combined with HIST features.

**Figure 5:**
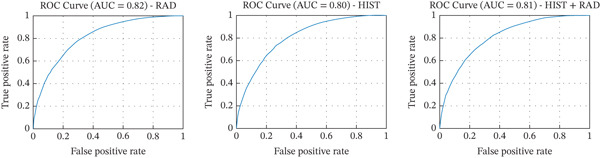
Receiver operating characteristic (ROC) curves for the three classification models. The curves show the trade‐off between true positive rate (sensitivity) and false positive rate (1—specificity). The area under the ROC curve (AUC) confirms the superior discriminative performance of the radiomic model.

Figure 5 shows the ROC curves for all three models. The radiomic model′s curve is always above the others, showing it can better classify tumor nuclei from nontumor nuclei. This is also confirmed by the AUC values: In fact, RAD (0.82) is higher than RAD + HIST (0.81) and HIST (0.80).

**Figure 6:**
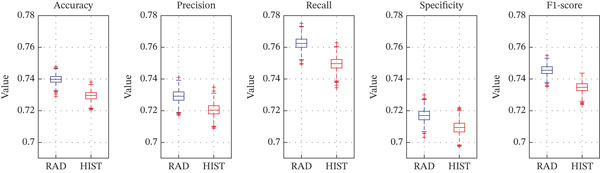
Bootstrap‐based boxplots showing the distribution of performance metrics across 1000 resampling iterations. The radiomic model boxplot is compared in terms of performance metrics (accuracy, precision, recall, F1‐score, and AUC), with the HIST model.

Furthermore, a bootstrap resampling strategy with 1000 iterations was executed to ensure that the performance differences observed were not due to random variations in training‐test. Bootstrap resampling allowed us to execute a statistical test to demonstrate the statistical significance of differences in metrics among the three models. In each iteration, models were retrained and evaluated on randomly resampled subsets, and all performance metrics were computed. The distribution of these metrics is presented in Figure 6, regarding the distributions of RAD and HIST model performances using boxplots. The RAD model shows tighter interquartile ranges and higher medians across all metrics. This observation indicates both robustness and stability of RAD features across different resampling iterations.

To statistically compare the performance metrics between models along the bootstrap iterations, the Wilcoxon signed‐rank test was used. The results are shown in Tables 2, 3, and 4. Table 2 compares the RAD and HIST models. All differences were statistically significant (*p* < 0.01), with the RAD model reaching better performances than the HIST model in every metric. Table 3 compares the HIST model and HIST + RAD model. Significant differences were observed for accuracy and F1‐score (*p* < 0.01), whereas other metrics did not show statistically significant differences, suggesting minimal benefit in combining the two feature types. Table 4 compares the RAD and RAD + HIST models. As already recorded for the comparison between RAD and HIST model, all metrics were significantly better for the RAD model (*p* < 0.01), confirming its superior performance even when compared with the combined model.

**Table 2:** Comparison between the RAD and HIST models across main performance metrics (mean ± standard deviation and 95*%*confidence interval). All differences are statistically significant (*p* < 0.01) based on the Wilcoxon signed‐rank test.

Metric	RAD (*m* *e* *a* *n* ± DS and IC95*%*)	HIST (*m* *e* *a* *n* ± DS and IC95*%*)	*p*value(*α* = 0.01)
Accuracy	0.7396 ± 0.0027 (IC95%: [0.7341, 0.7447])	0.7294 ± 0.0029 (IC95%: [0.7234, 0.7348])	*p* < 0.01
Precision	0.7291 ± 0.0039 (IC95%: [0.7213, 0.7368])	0.7205 ± 0.0039 (IC95%: [0.7128, 0.7283])	*p* < 0.01
Recall	0.7623 ± 0.0039 (IC95%: [0.7548, 0.7699])	0.7495 ± 0.0040 (IC95%: [0.7416, 0.7569])	*p* < 0.01
Specificity	0.7169 ± 0.0039 (IC95%: [0.7093, 0.7248])	0.7093 ± 0.0041 (IC95%: [0.7012, 0.7176])	*p* < 0.01
F1‐score	0.7454 ± 0.0031 (IC95%: [0.7391, 0.7509])	0.7347 ± 0.0032 (IC95%: [0.7282, 0.7406])	*p* < 0.01

**Table 3:** Comparison between the HIST and RAD+HIST models. Significant differences (*p* < 0.01) are observed for accuracy and F1‐score, whereas precision, recall, and specificity show no statistically significant differences (*p* > 0.01).

Metric	HIST (*m* *e* *a* *n* ± DS and IC95*%*)	*R* *A* *D* + *H* *I* *S* *T*(*m* *e* *a* *n* ± DS and IC95*%*)	*p*value(*α* = 0.01)
Accuracy	0.7294 ± 0.0029 (IC95%: [0.7234, 0.7348])	0.7292 ± 0.0028 (IC95%: [0.7236, 0.7344])	*p* < 0.01
Precision	0.7205 ± 0.0039 (IC95%: [0.7128, 0.7283])	0.7203 ± 0.0039 (IC95%: [0.7128, 0.7283])	**p** > 0.01
Recall	0.7495 ± 0.0040 (IC95%: [0.7416, 0.7569])	0.7493 ± 0.0039 (IC95%: [0.7413, 0.7570])	**p** > 0.01
Specificity	0.7093 ± 0.0041 (IC95%: [0.7012, 0.7176])	0.7092 ± 0.0040 (IC95%: [0.7016, 0.7170])	**p** > 0.01
F1‐score	0.7347 ± 0.0032 (IC95%: [0.7282, 0.7406])	0.7345 ± 0.0031 (IC95%: [0.7282, 0.7402])	*p* < 0.01

**Table 4:** Comparison between the RAD and RAD + HIST models. All performance metrics show statistically significant differences (p < 0.01) in favor of the radiomic model.

Metric	RAD (*m* *e* *a* *n* ± DS and IC95*%*)	*R* *A* *D* + *H* *I* *S* *T*(*m* *e* *a* *n* ± DS and IC95*%*)	*p*value(*α* = 0.01)
Accuracy	0.7396 ± 0.0027 (IC95%: [0.7341, 0.7447])	0.7292 ± 0.0028 (IC95%: [0.7236, 0.7344])	*p* < 0.01
Precision	0.7291 ± 0.0039 (IC95%: [0.7213, 0.7368])	0.7203 ± 0.0039 (IC95%: [0.7128, 0.7283])	*p* < 0.01
Recall	0.7623 ± 0.0039 (IC95%: [0.7548, 0.7699])	0.7493 ± 0.0039 (IC95%: [0.7413, 0.7570])	*p* < 0.01
Specificity	0.7169 ± 0.0039 (IC95%: [0.7093, 0.7248])	0.7092 ± 0.0040 (IC95%: [0.7016, 0.7170])	*p* < 0.01
F1‐score	0.7454 ± 0.0031 (IC95%: [0.7391, 0.7509])	0.7345 ± 0.0031 (IC95%: [0.7282, 0.7402])	*p* < 0.01

Figure 7 presents the distributions of the performance metrics across bootstrap iterations for RAD model versus RAD + HIST model (Figure 7a) and HIST model versus RAD + HIST hybrid model (Figure 7b). The plots visually confirm the statistical findings about a higher central tendency and a narrower spread for the RAD classifier that behaves consistently across resampling variations. Thus, RAD features extracted through PyRadiomics obtained better performance than the traditional HIST descriptors in classifying tumor nuclei from WSIs in melanoma. Furthermore, the hybrid model was not helpful for improving classification performance, meaning that RAD features alone are sufficient and more robust for this classification task.

Figure 7Bootstrap‐based boxplots showing the distribution of performance metrics across 1000 resampling iterations. The RAD model boxplot is compared in terms of performance metrics (accuracy, precision, recall, F1‐score, and AUC), with the HIST model (a). The HIST model is compared with the RAD + HIST hybrid model (b).

**Figure:**
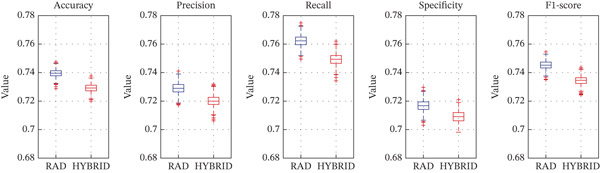
(a)

**Figure:**
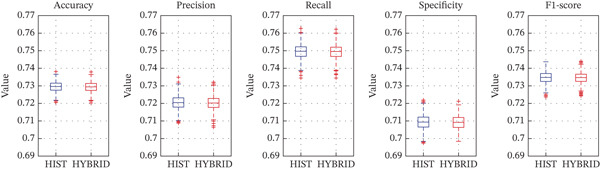
(b)

## 5. Discussion

In this study, the potential of RAD features extracted through PyRadiomics for the classification of tumor nuclei in melanoma histopathological WSIs was investigated relying on the publicly available PUMa dataset. The comparative analysis showed that, within the adopted experimental setup and default configurations, the classifier trained on PyRadiomics features achieved higher performance than the one based on HistomicsTK ones in all evaluated performance metrics: accuracy, precision, recall, specificity, F1‐score, and AUC. However, these findings must be interpreted as an empirical observation specific to the PUMa melanoma dataset and the binary classification task addressed. To statistically validate these results, bootstrap resampling and statistical comparisons were executed among the three models. Statistical results indicated that, under the adopted default configurations, the RAD model exhibited higher robustness and performance. The whole methodology was developed to ensure fair comparisons among the three models through the use of the same data splits, consistent LASSO feature selection, class balancing through under sampling of the majority class and the evaluation with bootstrap resampling. One of the most remarkable findings is that the RAD model, provided by PyRadiomics features in their default configuration, offered stronger discriminative power in this dataset compared with the nuclei‐oriented descriptors extracted with HistomicsTK. It must be noted, however, that PyRadiomics and HistomicsTK do not extract equivalent feature families. More specifically, PyRadiomics includes richer and more systematic texture descriptors, whereas HistomicsTK emphasizes morphology. As a result, the comparison partly reflects feature availability rather than feature effectiveness. Therefore, the observed performance differences may arise from these differences in feature catalogues and implementation choices, rather than solely from a conceptual contrast between pixel‐level and object‐level representations. This suggests that some intensity‐ and texture‐related feature families available in PyRadiomics captured signals that were particularly informative for this classification task. Specifically, the selection of texture features (such as nonuniformity) likely reflects the irregular distribution of chromatin and the structural variations within the nucleus, which are common signs of malignancy. In particular, the RAD features retained by the hybrid model provide a biologically interpretable description of melanoma tumor nuclei. First‐order intensity descriptors such as *“original_firstorder_10Percentile”* and *“original_firstorder_Minimum”* reflect the presence of low‐intensity regions within the nucleus, which are often associated with hyperchromatic regions in H&E‐stained malignant nuclei and increased DNA content. Texture‐based features derived from GLDM and GLRLM matrices, including *“original_gldm_DependenceVariance*,” *“original_gldm_GrayLevelNonUniformity*,” *“original_glrlm_RunLengthNonUniformityNormalized*,” and *“original_glrlm_ShortRunEmphasis,*” capture spatial heterogeneity and local intensity fluctuations, indicative of irregular chromatin organization and loss of nuclear structural uniformity. These alterations can be interpreted as signs of nuclear atypia in melanoma and support the biological relevance of the features selected by the LASSO‐based hybrid model.

The results align with the findings of Radulović et al. [22], confirming that RAD‐based textural analysis is a valuable tool for quantitative histopathology. However, although their work primarily focuses on tissue‐level patterns, the current study focuses on a cellular‐level exploration. This focus on individual nuclei also distinguishes our approach from the work of Linares et al. [23], who utilized RAD for broader tissue classification. Furthermore, although Sheikh and Cho [21] applied RAD to improve nuclei segmentation, our findings our study shifts the focus on a classification task. This work contributes to this growing research field through a melanoma‐based workflow. It is important to clarify that this study compares the empirical behavior of two feature‐extraction toolkits, PyRadiomics and HistomicsTK, rather than contrasting RAD and pathomics as conceptual domains. The hybrid RAD + HIST model, based on both statistically selected RAD and HIST features, has been evaluated to assess the potential combination of these descriptors in tumor nuclei classification. However, the model did not result in significant improvement of performance if compared with the RAD‐only model. This result suggests that, in this specific setting, the two feature sets may provide overlapping information. In fact, the lack of synergy suggests that the HistomicsTK descriptors provided redundant information already covered by PyRadiomics. Adding these correlated features increased the complexity of the model without providing new discriminative power, potentially introducing noise that limited the classifier′s efficiency. Even if promising results were shown, several limitations must be acknowledged. First, the study was conducted on a specific melanoma dataset, the PUMa dataset, on a task‐specific binary nuclei classification. To enhance the approach′s generalizability, an external validation on an independent melanoma WSIs is needed; further studies on different tissues and staining conditions are needed as well to assess the generalizability of these findings to broader computational pathology. Secondly, PyRadiomics provides a reliable feature extraction framework compliant with IBSI guidelines; however, it does not consider the tissue context or spatial relationships among nuclei. In addition, the simple binary classification of tumor versus nontumor nuclei underestimates the complicated biological spectrum. Future work could incorporate stromal features, nuclear–cytoplasmic ratios, and spatial interactions to better capture tumor ecosystem heterogeneity. In fact, the next stage of this work may investigate a multiclass approach or even hierarchical models to represent the tumor microenvironment. Moreover, a future extension will analyze RAD feature distributions separately in primary and metastatic melanoma, considering the evolutionary dynamics described in recent literature [39]. Furthermore, patch‐level RAD analyses, as used in recent studies [21], could be explored as a complementary approach by considering a broader tissue region to nuclei feature extraction in WSIs. Finally, although DL represents the current mainstream in computational pathology, its implementation was considered beyond the scope of this investigation. The decision to employ a feature‐based approach was driven by the objective to prioritize interpretability and to establish a computationally lightweight benchmark. Unlike DL architectures, which typically need massive datasets and significant computing power to avoid overfitting, handcrafted descriptors allow for a more direct control over the feature space, making them suitable for constrained data scenario. This work establishes a necessary baseline, offering explainable morphological markers that can complement future DL‐based studies. In future, neural networks trained on RAD features could further improve classification performance. By involving both the explainability of AI and the features, trust and understanding in clinical settings might be improved.

## 6. Conclusions

In this study, two commonly used feature‐extraction toolkits, PyRadiomics and the nuclei‐oriented HistomicsTK pipeline, were empirically compared when applied to the classification of tumor and nontumor nuclei in melanoma WSIs from the PUMa dataset. Using consistent preprocessing, identical training/test partitions, and a unified feature‐selection and evaluation framework with default settings, the random forest classifier trained on PyRadiomics features achieved higher performance than the models based on HistomicsTK features alone or on their combination for the specific task of binary nuclei classification within the PUMa melanoma dataset. These results suggest that, under default settings and within this task‐specific experimental framework, the feature set generated by PyRadiomics yielded higher discriminative performance than the nuclei‐specific features extracted via HistomicsTK. Meanwhile, the methodological framework employed here through feature selection, model evaluation, and statistical confirmation provides the basis for the integrity and replicability of the results.

The findings should not be interpreted as a conceptual comparison between “radiomics” and “pathomics,” which share common methodological foundations, but rather as a practical investigation of how two established toolkits behave on single‐nucleus classification when used with their standard configurations. The outcome also highlights that combining the two feature families did not yield additional benefit over RAD alone, possibly reflecting redundancy between feature sets or the strong contribution of intensity‐based descriptors for this task.

Future developments of this study will focus on validating the proposed technique with the use of external datasets, broadening the classification to multiclass issues. The analysis will also be extended to multiclass nuclei categorization, to investigate whether contextual, stromal, and spatial features can enhance nuclei‐level characterization, and it will be assessed how RAD and DL models can be integrated to improve performance and interpretability.

## Author Contributions

Alessia Finti and Franco Marinozzi share first authorship.

## Funding

No funding was received for this manuscript.

## Conflicts of Interest

The authors declare no conflicts of interest.

## Data Availability

The histopathology images and annotations used in this study are publicly available as part of the PUMa dataset, released for the “PUMa: Public Multi‐center histopathology dataset for automatic nuclei segmentation and classification” Grand Challenge. The dataset can be accessed at https://puma.grand-challenge.org/dataset/. All data were used in accordance with the terms and conditions of the original release.

## References

[1] World Health Organization , Skin Cancer, 2025, International Agency for Research on Cancer (IARC), https://www.iarc.who.int/cancer-type/skin-cancer.

[2] De Pinto G. , Mignozzi S. , La Vecchia C. , Levi F. , Negri E. , and Santucci C. , Global Trends in Cutaneous Malignant Melanoma Incidence and Mortality, Melanoma Research. (2024) 34, 265–275, 10.1097/CMR.0000000000000959. 38391175 PMC11045545

[3] Boutros A. , Croce E. , Ferrari M. , Gili R. , Massaro G. , Marconcini R. , Arecco L. , Tanda E. T. , and Spagnolo F. , The Treatment of Advanced Melanoma: Current Approaches and New Challenges, Critical Reviews in Oncology/Hematology. (2024) 196, 104276, 10.1016/j.critrevonc.2024.104276. 38295889

[4] Sundararajan S. , Thida A. M. , Yadlapati S. , Mukkamalla S. K. R. , and Koya S. , Metastatic Melanoma, 2025, StatPearls Publishing. 29262232

[5] Hsieh M. , Hsu S.-K. , Liu T.-Y. , Wu C.-Y. , and Chiu C.-C. , Melanoma Biology and Treatment: A Review of Novel Regulated Cell Death-Based Approaches, Cancer Cell International. (2024) 24, 10.1186/s12935-024-03220-9. PMC10858604 38336727

[6] de Paula Alves Coelho K. M. , de Macedo M. P. , Lellis R. F. , de Pinheiro-Junior N. F. , Rocha R. F. , Xavier-Junior J. C. , Dermatopathology Committee of the Brazilian Society of Pathology , and Paulo S. , Guidelines for Diagnosis and Pathological Report of Melanocytic Skin Lesions Recommendations From the Brazilian Society of Pathology, Surgical and Experimental Pathology. (2025) 8, 10.1186/s42047-025-00178-4.

[7] Pavlidis E. T. and Pavlidis T. E. , Diagnostic Biopsy of Cutaneous Melanoma, Sentinel Lymph Node Biopsy and Indications for Lymphadenectomy, World Journal of Clinical Oncology. (2022) 13, 861–865, 10.5306/wjco.v13.i10.861. 36337309 PMC9630995

[8] van Timmeren J. E. , Cester D. , Tanadini-Lang S. , Alkadhi H. , and Baessler B. , Radiomics in Medical Imaging—“How-To” Guide and Critical Reflection, Insights into Imaging. (2020) 11, 10.1186/s13244-020-00887-2. PMC7423816 32785796

[9] Amrane K. , Meur C. L. , Thuillier P. , Berthou C. , Uguen A. , Deandreis D. , Bourhis D. , Bourbonne V. , and Abgral R. , Review on Radiomic Analysis in 18F-Fluorodeoxyglucose Positron Emission Tomography for Prediction of Melanoma Outcomes, Cancer Imaging. (2024) 24, 10.1186/s40644-024-00732-5. PMC11225300 38970050

[10] Peisen F. , Hänsch A. , Hering A. , Brendlin A. S. , Afat S. , Nikolaou K. , Gatidis S. , Eigentler T. , Amaral T. , Moltz J. H. , and Othman A. E. , Combination of Whole-Body Baseline CT Radiomics and Clinical Parameters to Predict Response and Survival in a Stage-IV Melanoma Cohort Undergoing Immunotherapy, Cancers. (2022) 14, 10.3390/cancers14122992. PMC9221470 35740659

[11] McGale J. , Hama J. , Yeh R. , Vercellino L. , Sun R. , Lopci E. , Ammari S. , and Dercle L. , Artificial Intelligence and Radiomics: Clinical Applications for Patients With Advanced Melanoma Treated With Immunotherapy, Diagnostics. (2023) 13, 10.3390/diagnostics13193065. PMC10573034 37835808

[12] Stefano A. , Challenges and Limitations in Applying Radiomics to PET Imaging: Possible Opportunities and Avenues for Research, Computers in Biology and Medicine. (2024) 179, 108827, 10.1016/j.compbiomed.2024.108827. 38964244

[13] Bauckneht M. , Pasini G. , Di Raimondo T. , Russo G. , Raffa S. , Donegani M. I. , Dubois D. , Peñuela L. , Sofia L. , Celesti G. , Bini F. , Marinozzi F. , Lanfranchi F. , Laudicella R. , Sambuceti G. , and Stefano A. , [18F]PSMA-1007 PET/CT-Based radiomics May Help Enhance the Interpretation of Bone Focal Uptakes in Hormone-Sensitive Prostate Cancer Patients, European Journal of Nuclear Medicine and Molecular Imaging. (2025) 52, 2076–2086, 10.1007/s00259-025-07085-6. 39873702 PMC12014812

[14] Gupta R. , Kurc T. , Sharma A. , Almeida J. S. , and Saltz J. , The Emergence of Pathomics, Current Pathobiology Reports. (2019) 7, 73–84, 10.1007/s40139-019-00200-x, 2-s2.0-85069829006.

[15] Wu Y. , Li Y. , Xiong X. , Liu X. , Lin B. , and Xu B. , Recent Advances of Pathomics in Colorectal Cancer Diagnosis and Prognosis, Frontiers Oncology. (2023) 13, 1094869, 10.3389/fonc.2023.1094869. PMC10396402 37538112

[16] Zhang W. , Yang S. , Luo M. , He C. , Li Y. , Zhang J. , Wang X. , and Wang F. , Keep It Accurate and Robust: An Enhanced Nuclei Analysis Framework, Computational and Structural Biotechnology Journal. (2024) 24, 699–710, 10.1016/j.csbj.2024.10.046. 39650700 PMC11621583

[17] Lee S. , Amgad M. , Mobadersany P. , McCormick M. , Pollack B. P. , Elfandy H. , Hussein H. , Gutman D. A. , and Cooper L. A. , Interactive Classification of Whole-Slide Imaging Data for Cancer Researchers, Cancer Research. (2021) 81, 1171–1177, 10.1158/0008-5472.CAN-20-0668. 33355190 PMC8026494

[18] Pourakpour F. , Szölgyén Á. , Nateghi R. , Gutman D. A. , Manthey D. , and Cooper L. A. , HistomicsTK: A Python Toolkit for Pathology Image Analysis Algorithms, SoftwareX. (2025) 31, 102318, 10.1016/j.softx.2025.102318. 41048402 PMC12494233

[19] Sannachi L. , Mohabir S. , McNabb E. , Sharma D. , Giles A. , Yang W. , Leong K. X. , Stanisz M. , and Czarnota G. J. , Texture Analysis of Histology Images for Characterizing Ultrasound-Stimulated Microbubble Radiation Enhancement Treatment Response, Cells. (2025) 14, 10.3390/cells14242023. PMC12731841 41440043

[20] Lu C. , Shiradkar R. , and Liu Z. , Integrating Pathomics With Radiomics and Genomics for Cancer Prognosis: A Brief Review, Chinese Journal of Cancer Research. (2021) 33, 563–573, 10.21147/j.issn.1000-9604.2021.05.03. 34815630 PMC8580801

[21] Sheikh T. S. and Cho M. , Segmentation of Variants of Nuclei on Whole Slide Images by Using Radiomic Features, Bioengineering. (2024) 11, 10.3390/bioengineering11030252. PMC10967723 38534526

[22] Radulović M. , Li X. , Djuričić G. J. , Milovanović J. , Todorović Raković N. , Vujasinović T. , Banovac D. , and Kanjer K. , Bridging Histopathology and Radiomics Toward Prognosis of Metastasis in Early Breast Cancer, Microscopy and Microanalysis. (2024) 30, 751–758, 10.1093/mam/ozae057. 38973606

[23] Linares O. C. , Belizario I. V. , Batah S. S. , Hamann B. , Fabro A. T. , Azevedo-Marques P. M. , and Traina A. J. M. , RadPleura: A Radiomics-Based Framework for Lung Pleura Classification in Histology Images From Interstitial Lung Diseases, 2024, 2024 IEEE International Symposium on Biomedical Imaging (ISBI), 10.1109/ISBI56570.2024.10635328.

[24] Dia A. K. , Ebrahimpour L. , Yolchuyeva S. , Tonneau M. , Lamaze F. C. , Orain M. , Coulombe F. , Malo J. , Belkaid W. , Routy B. , Joubert P. , Després P. , and Manem V. S. K. , The Cross-Scale Association Between Pathomics and Radiomics Features in Immunotherapy-Treated NSCLC Patients: A Preliminary Study, Cancers. (2024) 16, 10.3390/cancers16020348. PMC10813866 38254838

[25] Brancato V. , Garbino N. , Aiello M. , Salvatore M. , and Cavaliere C. , Exploratory Analysis of Radiomics and Pathomics in Uterine Corpus Endometrial Carcinoma, Scientific Reports. (2024) 14, 10.1038/s41598-024-78987-y. PMC11680997 39730425

[26] Kim R. H. , Nomikou S. , Coudray N. , Jour G. , Dawood Z. , Hong R. , Esteva E. , Sakellaropoulos T. , Donnelly D. , Moran U. , Hatzimemos A. , Weber J. S. , Razavian N. , Aifantis I. , Fenyo D. , Snuderl M. , Shapiro R. , Berman R. S. , Osman I. , and Tsirigos A. , Deep Learning and Pathomics Analyses Reveal Cell Nuclei as Important Features for Mutation Prediction of BRAF-Mutated Melanomas, Journal of Investigative Dermatology. (2022) 142, 1650–1658, 10.1016/j.jid.2021.09.034. 34757067 PMC9054943

[27] Faust K. , Chen M. L. , Zadeh P. B. , Oreopoulos D. , Leon A. J. , Kamski-Hennekam E. R. , Mikhail M. , Duan X. , Duan X. , Liu M. , Ahangari N. , Cotau R. , Castillo V. F. , Nikzad N. , Sugden R. J. , Murphy P. , Done S. , Aljohani S. S. , Echelard P. , Jakate K. , Alwelaie Y. , Alyousef M. J. , Alsafwani N. S. , Alrumeh A. S. , Saleeb R. , Richer M. , Marins L. V. , Yousef G. M. , and Diamandis P. , PHARAOH: A Collaborative Crowdsourcing Platform for PHenotyping And Regional Analysis of Histology, BioRxiv. (2025) 16, 10.1101/2024.03.20.585977. PMC11739387 39820318

[28] Li M. , Abe M. , Nakano S. , and Tsuneki M. , Deep Learning Approach to Classify Cutaneous Melanoma in a Whole Slide Image, Cancers. (2023) 15, 10.3390/cancers15061907. PMC10047087 36980793

[29] Tripathi S. and Singh S. K. , Ensembling Handcrafted Features With Deep Features: An Analytical Study for Classification of Routine Colon Cancer Histopathological Nuclei Images, Multimedia Tools and Applications. (2020) 79, 34931–34954, 10.1007/s11042-020-08891-w.

[30] Huang X. , Li Z. , Zhang M. , and Gao S. , Fusing Hand-Crafted and Deep-Learning Features in a Convolutional Neural Network Model to Identify Prostate Cancer in Pathology Images, Frontiers in Oncology. (2022) 12, 994950, 10.3389/fonc.2022.994950. 36237311 PMC9552083

[31] Akbarpour M. , Fazlollahiaghamalek H. , Barati M. , Kamangar M. H. , and Mandal M. , Deep Learning-Based Nuclei Segmentation and Melanoma Detection in Skin Histopathological Image Using Test Image Augmentation and Ensemble Model, Journal of Imaging. (2025) 11, 10.3390/jimaging11080274. PMC12387607 40863484

[32] Van Griethuysen J. J. M. , Fedorov A. , Parmar C. , Hosny A. , Aucoin N. , Narayan V. , Beets-Tan R. G. H. , Fillion-Robin J.-C. , Pieper S. , and Aerts H. J. W. L. , Computational Radiomics System to Decode the Radiographic Phenotype, Cancer Research. (2017) 77, e104–e107, 10.1158/0008-5472.can-17-0339, 2-s2.0-85035021353. 29092951 PMC5672828

[33] HistomicsTK , Manage, Analyze, and Visualize Your Digital Pathology Data, 2024, HistomicsTK, https://histomicstk.kitware.com/.

[34] Schuiveling M. , Melanoma Histopathology Dataset With Tissue and Nuclei Annotations, 2025, Zenodo, https://zenodo.org/records/15050523.

[35] Schuiveling M. , Liu H. , Eek D. , and Breimer G. E. , A Novel Dataset for Nuclei and Tissue Segmentation in Melanoma With Baseline Nuclei Segmentation and Tissue Segmentation Benchmarks, GigaScience. (2025) 14, 10.1093/gigascience/giaf011. PMC11837757 39970004

[36] Zwanenburg A. , Vallières M. , Abdalah M. A. , Aerts H. J. W. L. , Andrearczyk V. , Apte A. , Ashrafinia S. , Bakas S. , Beukinga R. J. , Boellaard R. , Bogowicz M. , Boldrini L. , Buvat I. , Cook G. J. R. , Davatzikos C. , Depeursinge A. , Desseroit M. C. , Dinapoli N. , Dinh C. V. , and Echegaray S. , The Image Biomarker Standardization Initiative: Standardized Quantitative Radiomics for High-Throughput Image-Based Phenotyping, Radiology. (2020) 295, 328–338, 10.1148/radiol.2020191145. 32154773 PMC7193906

[37] HistomicsTK , Histomicstk Features, 2025, HistomicsTK, https://digitalslidearchive.github.io/HistomicsTK/histomicstk.features.html.

[38] MathWorks , MATLAB, 2025, MathWorks, https://uk.mathworks.com/products/matlab.html.

[39] Luo W. , Nasopharyngeal Carcinoma Ecology Theory: Cancer as Multidimensional Spatiotemporal “Unity of Ecology and Evolution” pathological Ecosystem, Theranostics. (2023) 13, no. 5, 1607–1631, 10.7150/thno.82690. 37056571 PMC10086202

